# Amorphous phase-change memory alloy with no resistance drift

**DOI:** 10.1038/s41563-025-02361-0

**Published:** 2025-10-01

**Authors:** Xiaozhe Wang, Ruobing Wang, Suyang Sun, Ding Xu, Chao Nie, Zhou Zhou, Chenyu Wen, Junying Zhang, Ruixuan Chu, Xueyang Shen, Wen Zhou, Zhitang Song, Jiang-Jing Wang, En Ma, Wei Zhang

**Affiliations:** 1 Center for Alloy Innovation and Design (CAID), State Key Laboratory for Mechanical Behavior of Materials, Xi’an Jiaotong University, Xi’an, China; 2 National Key Laboratory of Materials for Integrated Circuits, Shanghai Institute of Microsystem and Information Technology, Chinese Academy of Sciences, Shanghai, China

**Keywords:** Materials science, Metals and alloys

## Abstract

Spontaneous structural relaxation is intrinsic to glassy materials due to their metastable nature. For phase-change materials, the resultant temporal change in electrical resistance seriously hampers neuromorphic computing applications. Here we report an ab-initio-calculation-informed design of amorphous phase-change materials composed of robust ‘molecule-like’ motifs, depriving the amorphous alloy of critical structural ingredients responsible for relaxation and, hence, resistance drift. We demonstrate amorphous CrTe_3_ thin films that display practically no resistance drift at any working temperature from −200 °C to 165 °C, and highlight the multilevel encoding ability via a hybrid opto-electronic approach. We further reveal that the same no-drift behaviour holds for melt-quenched amorphous CrTe_3_ in electronic devices. Moreover, the application potential of CrTe_3_ is testified by its incorporation in a vehicle with an automatic path-tracking function. Our work provides an alternative route to achieve requisite properties for potential phase-change neuromorphic computing via the judicious design of disordered phase-change materials.

## Abstract (web summary)

Resistance drift, also known as the temporal change in electrical resistance, hampers the application of phase-change materials for neuromorphic computing. Here an amorphous CrTe_3_ thin film with no resistance drift in the working temperature from −200 °C to 165 °C is reported.

## Main

Data-intensive applications, such as artificial intelligence and the Internet of Things, are having a profound impact on nearly all aspects of life. The huge amount of data being generated at an ever-increasing rate poses formidable challenges to efficient data storage and processing. Neuromorphic computing or in-memory computing (IMC) based on resistive-switching non-volatile memory arrays, unifying storage with computing at the same location, can substantially improve the computing efficiency at a much lower energy cost^1–4^. The key to high-storage-density neuromorphic computing is to obtain as many distinguishable resistance levels as possible in every memory/computing cell^5^. Yet, high-precision IMC is facing a bottleneck problem associated with the intrinsic randomness of materials on programming^6^, especially when operated at a working temperature (*T*) different from room temperature (RT). Continuous cooling is feasible at power farms, but for the power-limited edge-computing platforms, temperature effects^7–9^ on accurate multilevel programming are much more critical.

Chalcogenide phase-change materials (PCMs), which utilize the large resistance contrast between their amorphous and crystalline states for data encoding^10,11^, are one of the leading candidates for non-volatile memories. The flagship Ge_2_Sb_2_Te_5_ (GST) alloys with moderate doping^12^ or heavy alloying^13^ have enabled the industrial production of high-density three-dimensional cross-point memory cards for computing servers and data centres with the operating temperature from ~60 °C to 80 °C, as well as embedded memory units for automotive with a working temperature range from approximately −40 °C to 165 °C (ref. ^14^). Very recently, it has also been shown that crystalline PCMs and amorphous PCMs (a-PCMs) can survive the harsh space environment outside of the International Space Station^15^, where temperature can cycle between −120 °C and 120 °C sixteen times per day. Beyond the success in these binary storage applications, PCMs do show multilevel storage capacity. However, the spontaneous structural relaxation—aging—of a-PCM causes intolerable resistance drift^16–18^. This is a pressing problem threatening the device accuracy needed for neuromorphic computing applications^19^.

Massive research efforts have been devoted to overcoming the adverse impact of resistance drift. On the device side, several approaches using mixed-precision computing^19^, algorithm compensation^20^, metal liner confinement^21^ and multi-PCM units^22^ were developed to partially mitigate the drift problem. It remains unclear whether these workaround solutions can still function at elevated temperatures or on extreme temperature cycling, but in any case, they add complexities to high-density device manufacturing and accurate multilevel programming. Therefore, a fundamental solution on the materials side, that is, PCMs with intrinsically low drift, is sorely needed. Previous attempts using impurity doping, heterostructure confinement and downscaling the film thickness only managed to reduce the drift tendency at RT^23–29^. However, there is so far no candidate PCM that can generate many robust resistance states to function across a wide range of operating temperatures for various application scenarios. In the following, we offer a materials solution to diminish relaxation/drift once and for all, via a paradigm shift towards ‘molecular-glass-like’ a-PCMs. This approach enabled us to discover an a-PCM that is intrinsically invulnerable to resistance drift at all working temperatures practically encountered in IMC operations.

## Design of molecular-glass-like a-PCM

**Fig. 1:**
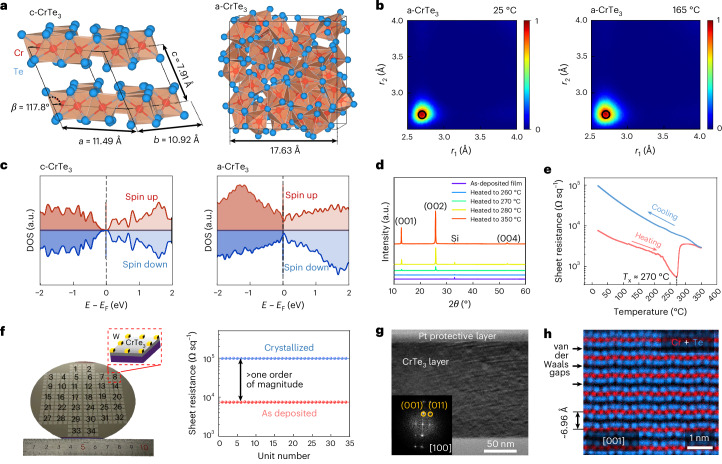
Materials design, synthesis and characterizations. **a**, Relaxed atomic structures of c-CrTe_3_ and melt-quenched a-CrTe_3_. The Cr and Te atoms are rendered with red and light blue spheres and the [CrTe_6_] octahedra are highlighted by orange polyhedra. **b**, ALTBC plot of a-CrTe_3_ showing no Peierls distortion at ~25 °C and ~165 °C. The black circle indicates equal bonds in c-CrTe_3_ (~2.7 Å). **c**, Calculated DOS of c-CrTe_3_ and a-CrTe_3_. **d**, XRD patterns of the as-deposited thin film and the thin films heated to 260 °C, 270 °C, 280 °C and 350 °C. **e**, Temperature dependence of the sheet resistance of a CrTe_3_ film heated up to 350 °C at a heating rate of 10 °C min^− 1^, and was then cooled to RT. **f**, van der Paul measurement of 34 units on a four-inch wafer. The 0.5 × 0.5 cm^2^ CrTe_3_ area in the middle of the substrate is contacted by eight tungsten electrodes (marked in yellow), which were patterned at the edges of the 1.2 × 1.2 cm^2^ substrate. **g**,**h**, TEM bright-field image (**g**) and the atomic-scale elemental mapping (**h**) of the c-CrTe_3_ film that was heated to 350 °C. The positions of the Te and Cr columns are marked in blue and red, respectively. The thickness of the films was ~150 nm for structural characterizations and ~50 nm for electrical measurements.

On aging, a-PCMs are prone to structural relaxation. Conventional a-PCMs experience a gradual (1) reinforcement of Peierls distortion of (defective) octahedral motifs^30^ and (2) disappearance with increasing time (*t*) of salient structural defects^30–32^, including ‘wrong’ bonds (for example, Ge−Ge homopolar bonds) and tetrahedral motifs. Both these factors result in resistance drift. Our key idea is then to find an a-PCM that intrinsically has no such tendencies during time- and temperature-induced relaxation (Supplementary Note 1 and Supplementary Fig. 1). Figure 1a shows relaxed crystalline CrTe_3_ (c-CrTe_3_) calculated via spin-polarized density functional theory (DFT). First, the directional bonds of c-CrTe_3_ show no obvious Peierls distortion; the bond lengths are nearly identical, and the maximum difference is as small as 0.03 Å, an order of magnitude smaller than that of rhombohedral GeTe (~0.4 Å). Second, the crystal shows a layered structure composed exclusively of corner- and edge-sharing [CrTe_6_] octahedra. It is, thus, possible that its amorphous phase (a-CrTe_3_) would also contain a very high density of non-defective [CrTe_6_] octahedra with little Peierls distortion. To see if this hypothesis is valid, we carried out DFT-based ab initio molecular dynamics (AIMD) calculations to obtain a-CrTe_3_ models of 200 atoms following a standard melt-quench approach. We first used the computed crystalline density directly, and the obtained atomic structure is shown in Fig. 1a. As highlighted using coordination polyhedra, almost all Cr atoms indeed form [CrTe_6_] octahedra (48 out of 50 Cr atoms, and the remaining two Cr octahedra have only one missing neighbour). This observation is further confirmed by quantitative structural analyses (Supplementary Fig. 2a).

We next evaluated the degree of Peierls distortion in the amorphous counterpart, that is, the a-CrTe_3_ model, by calculating the angular-limited three-body correlation (ALTBC) at RT (~25 °C) over directionally bonded pairs (bond angle, ~155°–180°). In stark contrast with conventional PCM with an extended wing shape^30^, a-CrTe_3_ displays a single primary peak around 2.72 Å in the ALTBC profile at RT (Fig. 1b), which matches that of the c-CrTe_3_ (black circle). We also calculated the ALTBC profiles of a-CrTe_3_ at other (lower and higher than RT) temperatures (Supplementary Fig. 2b), and they show a picture consistent with that at RT. Further optimization of the amorphous model at RT leads to a volume expansion by ~2.1%, but the structural features remain the same (Supplementary Fig. 2c). Four additional a-CrTe_3_ models in both crystalline and amorphous densities were calculated, which confirm that a-CrTe_3_ consists of [CrTe_6_] octahedra connected in a disordered fashion, without obvious Peierls distortion and structural defects. In fact, octahedra are the only local configuration observed, and these motifs can, hence, be deemed molecule-like (Supplementary Fig. 3).

With the desired structural features in hand, the next challenge is the requisite electronic properties. For IMC applications, it is imperative for the PCM to have a large contrast window in electrical resistance on switching. The computed electronic density of states (DOS) predicts a major difference in electronic structure between c-CrTe_3_ and a-CrTe_3_ (Fig. 1c). The crystalline phase is a semiconductor stabilized by antiferromagnetic interactions^33^. For the amorphous phase, we considered a couple of magnetic configurations, and the bandgap is always filled. Figure 1c shows the DOS for the ferromagnetic state, and other magnetic configurations are included in Supplementary Fig. 4. The open versus filled bandgaps should result in a sizable difference in electrical resistivity between the two states, with the amorphous state being the lower-resistance state, which was observed in a similar layered PCM, that is, CrGeTe_3_ (refs. ^34,35^). Importantly, as noted in Supplementary Fig. 2, the same octahedral configuration (‘molecules’ as well as their number density) stays predominant even when the temperature is elevated to 165 °C, suggesting that a-PCM should also have excellent thermal stability across a wide range of operating temperatures.

## Wafer-scale synthesis of high-quality CrTe_3_ films

**Extended Data Fig. 1:**
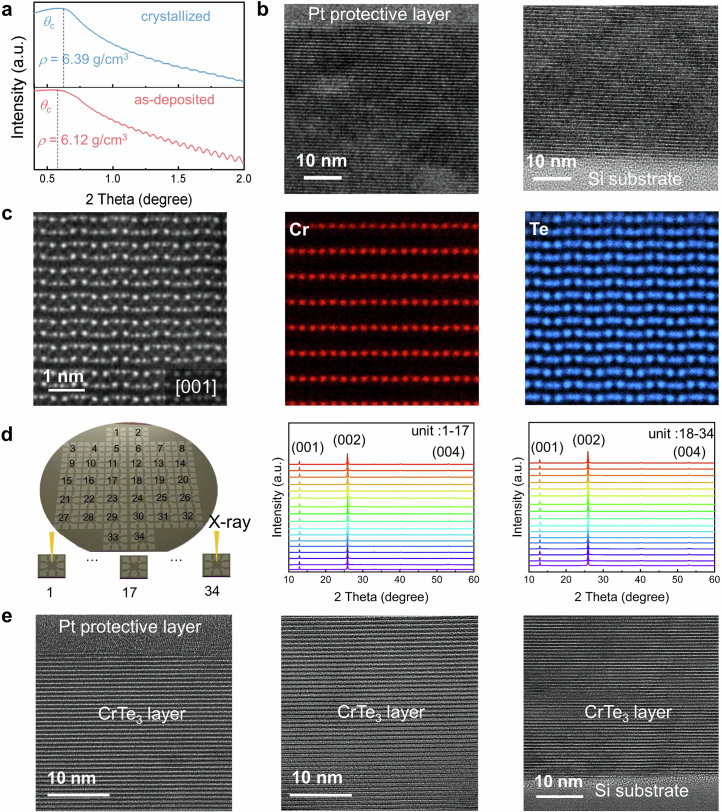
Structural characterizations of CrTe_3_ thin films. The samples were heated to 350 °C to obtain a completely crystallized state. (**a**) The XRR spectrum of as-deposited amorphous films and crystallized CrTe_3_ films of ~150 nm. (**b**) The TEM images near top Pt protective layer and the Si substrate of crystallized CrTe_3_ film of ~150 nm. The zoom-in TEM images taken at locations from the top to the bottom of the film consistently show well-aligned (001) lattice planes parallel to the substrate surface, consistent with the XRD results, indicating epitaxial-growth-like crystal quality even though no seeding layer was used in the deposition. (**c**) The atomic-scale HAADF image of crystalline CrTe_3_ viewed in the [001] direction and its corresponding EDX mapping of Cr and Te. The intensity of spots in the HAADF image is approximately proportional to the square of the averaged atomic number Z of each column along the view direction, therefore, the Te (Z = 54) columns look brighter than the Cr (Z = 24) columns. (**d**) The measured XRD patterns over 34 locations on the 4-inch wafer. (**e**) The TEM images recorded at different locations of crystallized CrTe_3_ film of ~50 nm. No clear grain boundary can be observed over >5 μm lateral direction. The film thickness along the beam incidence direction was ~80 nm for all TEM specimens we prepared.

We deposited CrTe_3_ films of ~50−150 nm in thickness on four-inch-diameter SiO_2_/Si substrates at RT, using a pure Cr target and a pure Te target via magnetron sputtering. For the as-deposited film and the one heated to 260 °C at a heating rate of 10 °C min^−1^, X-ray diffraction (XRD) patterns at RT (Fig. 1d) show no crystalline diffraction peaks, except for the silicon substrate. After heating to 270 °C, 280 °C and 350 °C, the (100), (200) and (400) peaks of c-CrTe_3_ appeared, indicating an out-of-plane texture. The lattice parameters obtained by XRD are 11.53 Å, 11.19 Å and 7.79 Å, in good agreement with the DFT calculation—11.49 Å, 10.92 Å and 7.91 Å, respectively. As estimated from X-ray reflectometry, the mass densities of the as-deposited amorphous and crystallized films were ~6.12 g cm^−3^ and ~6.39 g cm^−3^, respectively (Extended Data Fig. 1). Hence, on crystallization, a smaller increase in density (Δ*ρ*/*ρ*_crystal_) was observed for CrTe_3_ (~3.9%), compared with GST (~6.4%)^36^.

Next, we monitored the electrical resistance on the in situ heating of the as-deposited film to 350 °C at a heating rate of 10 °C min^−1^ using the van der Pauw method in an argon-protected environment. In agreement with our DFT prediction, Fig. 1e shows an inverse resistance contrast on crystallization for CrTe_3_, opposite to GST. The sheet resistance of the as-deposited amorphous film was ~7 kΩ sq^−1^ at RT, slowly and gradually decreasing on heating. Only after ~230 °C, the sheet resistance drops more quickly (Supplementary Fig. 5), reaching a minimum at *T*_x_ ≈ 270 °C, where crystallization started, which is consistent with the XRD data. The sheet resistance of the crystallized film was measured to be 100 kΩ sq^−1^ at RT. According to the Hall effect measurements, the carrier concentration and mobility of the as-deposited film were 5.1 × 10^20^ cm^−3^ and 6.8 × 10^−1^ cm^2^ V^−1^ s^−1^, respectively, which were reduced to 6.6 × 10^19^ cm^−3^ and 2.8 × 10^−1^ cm^2^ V^−1^ s^−1^, respectively, in the crystallized film. To assess the reproducibility, we fabricated 34 tungsten-electrode-based test cells, each containing eight electrodes, and measured the sheet resistances of the as-deposited amorphous phase and the fully crystallized phase at 350 °C. All 34 test cells gave highly consistent resistance values (Fig. 1f).

For the completely crystallized phase, the cross-sectional transmission electron microscopy (TEM) image in Fig. 1g looks homogeneous with limited intensity contrast, and no grain boundary can be observed at this magnification. The fast Fourier transform pattern confirms the crystal orientation of the c-CrTe_3_ film to be [001]. The atomic-scale elemental mapping images clearly show an arrangement of slabs of CrTe_3_ trilayers sandwiching van der Waals gaps (Fig. 1h). Additional TEM analyses confirmed the highly textured large grains across the four-inch c-CrTe_3_ film, and the electron-transparent region in the TEM foil (lateral dimension, >5 μm) of the 50-nm c-CrTe_3_ film was nearly a single grain (Extended Data Fig. 1). This structural uniformity leads to highly consistent resistance values across the whole silicon wafer (Fig. 1f).

## Suppressed resistance drift at extreme temperatures

**Fig. 2:**
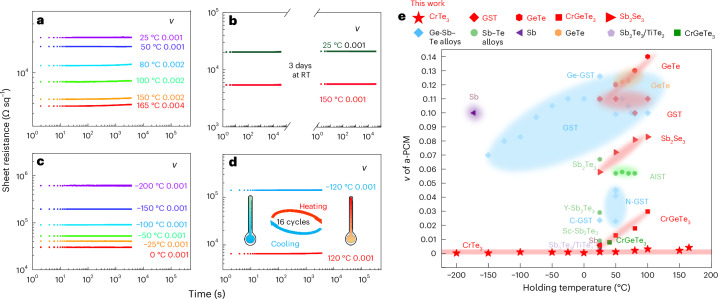
Minimal resistance drift. **a**, Resistance drift measurement of as-deposited a-CrTe_3_ films on heating over 1 h, each at a different holding temperatures. **b**, Two as-deposited a-CrTe_3_ films were measured at 25 °C and 150 °C over 10 h (first part, displayed on the left). After aging for 3 days at RT, they were measured at 25 °C and 150 °C over 10 h again (second part, on the right). **c**, Resistance of the a-CrTe_3_ film measured at 0 °C and below. **d**, The a-CrTe_3_ film was subjected to extreme temperature cycling between –120 °C and 120 °C over 16 times in 24 h, and subsequently, its sheet resistance was measured at –120 °C and 120 °C over time. The distinctive resistance levels were due to the varied thermally excited carrier concentrations at different temperatures. **e**, Summary of the measured drift coefficient of various a-PCM films. The drift coefficient is highly temperature dependent for other PCMs, for example, *ν* of a-Sb can be enlarged from ~0.001 at RT to ~0.1 at −173 °C.

The resistance drift behaviour of a-PCMs is usually characterized using *R*(*t*) = *R*_0_ (*t*/*t*_0_)^*ν*^ (*σ*(*t*) = *σ*_0_(*t*/*t*_0_)^−*ν*^), where *R*_0_ (*σ*_0_) is the resistance (conductance) measured at time *t*_0_, and *ν* is the drift coefficient over time *t*. To avoid potential aging effects due to sample storage, we measured the resistance of a-CrTe_3_ thin films immediately after their sputter preparation. We tested different film thicknesses from 50 nm to 150 nm, which showed an ultralow drift with *ν* ≈ 0.001 at RT. With a drift coefficient at this level, resistance drift would have little impact on practical multilevel programming. Figure 2a displays the as-deposited a-CrTe_3_ film (50 nm thick) heated to increasingly elevated temperatures, and at each temperature, the holding time was 1 h. The a-CrTe_3_ film showed a consistently low *ν* of 0.001−0.002 between RT and 150 °C. At 165 °C, its *ν* slightly increased to ~0.004. These values are far smaller than that of a-GST: *ν* ≈ 0.11 between RT and 100 °C (Supplementary Fig. 6). Another issue of a-GST is that it crystallizes quickly at higher temperatures (for example, when approaching 150 °C), making it unsuitable for embedded memory applications. Additional drift measurements were performed for a-GeTe, a-CrGeTe_3_ and a-Sb_2_Se_3_ thin films (Supplementary Fig. 6), which showed notable resistance drift.

The ultralow drift behaviour of a-CrTe_3_ was found to be robust over much longer times. We took two other freshly as-deposited films, and recorded their sheet resistance at 25 °C and 150 °C over 10 h. Then, the two films were stored for 3 days in air without argon protection at RT. Subsequently, the two films were measured again at 25 °C and 150 °C over another 10 h. Both sets of a-CrTe_3_ films showed *ν* ≈ 0.001 (Fig. 2b). We also recorded the resistance of a-CrTe_3_ at 0 °C and −25 °C, using our custom-made setup, and at even lower temperatures using a physical property measurement system with liquid-helium refrigeration. As shown in Fig. 2c, our a-CrTe_3_ film again displayed very steady resistance with little drift, *ν* ≈ 0.001, when held at any temperature from 0 °C down to −200 °C. We also confirmed that such minimal drift can be sustained on extreme temperature cycling (Fig. 2d).

**Extended Data Fig. 2:**
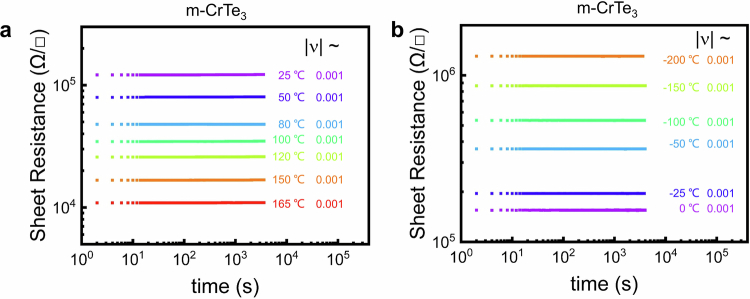
Electrical measurements of the crystallized thin films. The resistance drift measurements of the crystallized CrTe_3_ thin films, each conducted at a different holding temperature (**a**) at or above RT, and (**b**) at 0 °C or below.

Figure 2e summarizes the drift coefficient *ν* of a-CrTe_3_ (red stars) together with various a-PCMs^23–29,37–39^, plotted versus the holding temperature. Previous drift measurements of a-PCMs, including the best-so-far TiTe_2_/Sb_2_Te_3_ heterostructure^26^, were mostly conducted at RT. The holding temperature was limited to ~100 °C, due to their relatively low *T*_x_. Doping and alloying can increase the thermal stability of the amorphous phase, which, however, can also result in resistance drift in the crystalline phase, with *ν* reaching ~0.01 or even higher^39^. By contrast, for our c-CrTe_3_ (Extended Data Fig. 2), the sheet resistance showed a consistently low *ν* ≈ 0.001 at all the holding temperatures used for a-CrTe_3_. We also note that GeTe/Sb_2_Te_3_ and Sb_2_Te_3_/Ge-Sb-Te superlattices may display a relatively low drift of *ν* ≈ 0.002−0.009 (refs. ^40–42^). Nevertheless, their RESET states may be in a partly crystalline state. As shown in Fig. 2e, our CrTe_3_ is the only PCM that is immune to resistance drift at all practical operating temperatures for IMC applications.

## Electrical measurement of CrTe_3_ devices

**Fig. 3:**
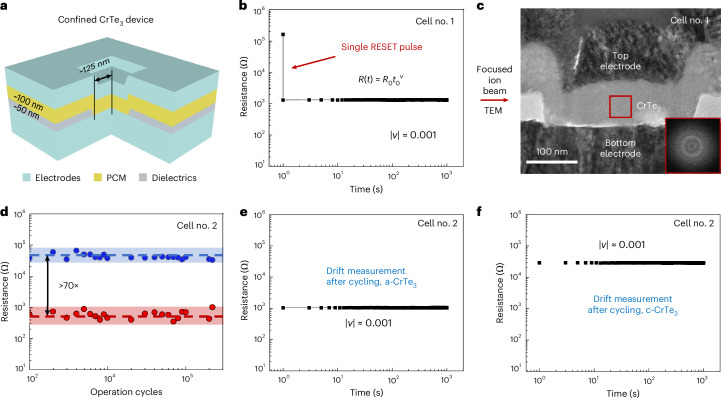
Electrical measurements using confined CrTe_3_ devices. **a**, Sketch of the confined electronic device with CrTe_3_ being the functional PCM layer. **b**, A single electrical pulse was applied to fully RESET a confined memory cell, after which its resistance was monitored immediately at RT. **c**, Subsequent cross-sectional TEM characterizations of the RESET state, proving that the cell turned fully amorphous (see halos in the fast Fourier transform pattern). **d**, Electrically switched cycling test of a second confined cell. **e**,**f**, Drift measurement at RT of a full RESET state (**e**) and a full SET state (**f**) after cycling.

**Extended Data Fig. 3:**
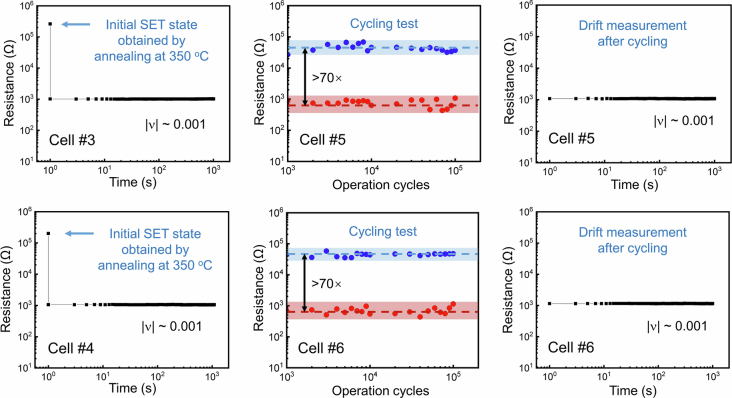
Additional drift measurements. The drift measurements after the initial electrical melt-quench RESET operation in electronic device, the cycling endurance test itself, and the drift measurements of the full RESET state after cycling at RT. Electrical measurements of four additional confined memory cells confirmed the no-drift behavior of the RESET state. See Supplementary Fig. 9 for the effect of pulse width in suppressing programming noise.

To ascertain that the melt-quenched a-CrTe_3_ displays the same no-drift behaviour, we carried out electrical measurements on a series of confined memory devices, inside which a CrTe_3_ film of ~100 nm is confined by the electrodes (tungsten) and the dielectric layers (SiO_2_; Fig. 3a). Further details about the device structure can be found in Supplementary Fig. 7. First, the CrTe_3_ cell was thermally annealed at 350 °C to form a full crystalline state, and was programmed to a full amorphous state using a single electrical RESET pulse (Fig. 3b). The drift coefficient (*ν* ≈ 0.001) determined from the data acquired immediately after the electrical melt-quench process (Supplementary Video 1) is consistent with the behaviour of the as-deposited amorphous phase. The subsequent cross-sectional TEM characterizations confirmed that the electrical switching rendered the cell fully amorphous (Fig. 3c). We also performed cycling measurements and found the same no-drift behaviour of the RESET state of a-CrTe_3_ after 2 × 10^5^ cycles (Fig. 3d,e). The same electrical measurements were repeated for several additional cells, and the drift coefficient values of the initial RESET and final RESET states are consistently close to 0.001 (Extended Data Fig. 3). Meanwhile, the SET state (that is, c-CrTe_3_) showed constant electrical resistance over time, too (Fig. 3f). Further results are documented in Supplementary Fig. 8. Note that the programming noise of the CrTe_3_ device can also be suppressed by increasing the pulsing width to reduce the crystallization randomness (Supplementary Fig. 9). Besides, we confirmed that consistently high resistance drift would persist for GST devices after extended cycling (Supplementary Fig. 10).

**Extended Data Fig. 4:**
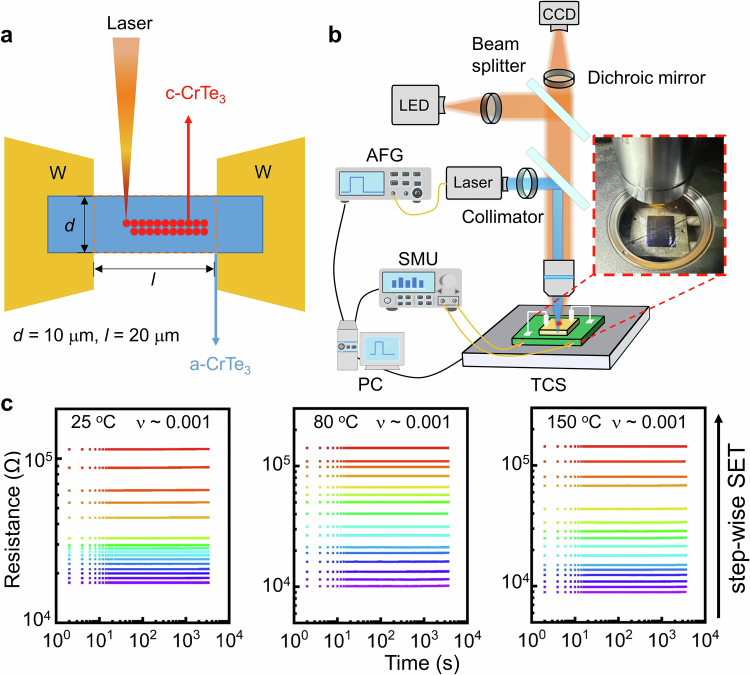
Deterministic multilevel programming. (**a**) The scheme of optical programming and electrical measurement on a bridge-like CrTe_3_ device. (**b**) The hybrid opto-electro-thermal setup consisted of a nanosecond laser beam with 1 μm spot size (controlled using an arbitrary functional generator, AFG), a source measure unit (SMU) for electrical measurements, and a temperature controller stage (TCS). The bottom-right inset shows the photo of the self-made CrTe_3_ device. (**c**) Using a bridge-like device of 10×20 μm^2^, 16 robust resistance states were obtained at 25, 80 and 150 °C via step-wise SET operations. Each resistance level was measured after sending two laser pulses.

The essence of multilevel programming in PCM devices is to generate a series of intermediate resistance states, each corresponding to a different ratio of amorphous-to-crystalline volume. However, fine-tuning the volume fraction of crystalline versus amorphous regions using electrical pulses requires sophisticated device fabrication and control, and is subject to much uncertainty. We, therefore, developed a hybrid opto-electro-thermal scheme, which serves the purpose of assessing the multilevel capacity of CrTe_3_ in a deterministic way. The precise volume control is achieved via step-wise SET, that is, generating crystalline domains of the same size, one by one in a regular pattern, via laser irradiation on an amorphous film. The actual image of the setup is displayed in Supplementary Fig. 11. Using a bridge-like device covered with an as-deposited a-CrTe_3_ film, we applied laser pulses in the middle part of the devices for sequential crystallization along the lateral direction (Supplementary Fig. 12 shows the optical images). After sending every two laser pulses, the electrical resistance was measured and monitored for over 1 h. During the whole process (>16 h), each of the three devices were held at 25 °C, 80 °C or 150 °C. Each device displayed 16 well-separated resistance levels (Extended Data Fig. 4). The resistance levels can be well distinguished even at a holding temperature of 165 °C (Supplementary Fig. 13). Although the crystallization rate is not a top-priority parameter to optimize owing to the parallel programming ability of the PCM array^43^, it is still desirable to have relatively fast crystallization to shorten the training time of the neural network (Supplementary Note 2 and Supplementary Fig. 14).

## Path-tracking function enabled by stable resistance levels

**Fig. 4:**
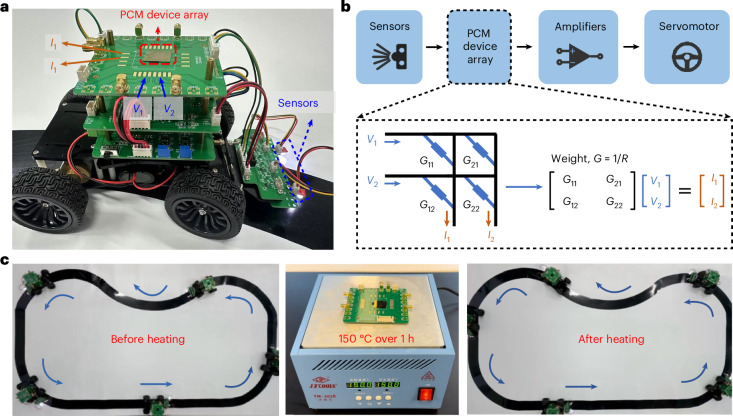
CrTe_3_-based path-tracking vehicle. **a**, Image of a CrTe_3_-based path-tracking vehicle. **b**, Schematic of the control system with a 2 × 2 CrTe_3_ device array. **c**, Path-tracking function was preserved after heating. Supplementary Video 2 shows the entire test process.

To highlight the importance of robust resistance levels without drift, we fabricated a 2 × 2 array of CrTe_3_-based bridge-like devices, and integrated the array into a self-made vehicle (Fig. 4a) to realize a stable path-tracking function. As shown in Fig. 4b, the control system of this vehicle mainly composed of two greyscale sensors, a device array, amplifiers and a servomotor. The sensors are symmetrically installed in the front of the vehicle to detect greyscale changes, which provide distinct input voltage signals when detecting black and white. We programmed the four well-separated resistance values into the four devices via step-wise SET laser operations on the as-deposited a-CrTe_3_ thin films. The weight (conductance) values of the four devices were then mapped into a single-layer neural network (the 2 × 2 array) as the basic neuromorphic computing unit, performing matrix vector multiplication through Ohm’s law and Kirchhoff’s law to enable automatic path tracking^44^. The output current signals from the array were then processed through the transimpedance amplifier and differential amplifier, and were converted into voltage signals again for the final steering and turning actions of the servomotor. The threshold for failure is set as tight as ‘any weight value changing by more than ±1%’. As shown in Fig. 4c and Supplementary Video 2, the vehicle appropriately tracked the black path, consistently turning the wheels back to the black path when detecting the white background. This CrTe_3_-based path-tracking vehicle still functioned very well after being placed in air for over 1 month, and after heating the control board at 150 °C for over 1 h. In a control experiment, a GST device array was integrated into the same vehicle. The automatic path-tracking function was achieved initially, but was completely lost after only 10 min (Supplementary Fig. 15 and Supplementary Video 3) due to the resistance drift of a-GST.

## Outlook

**Extended Data Fig. 5:**
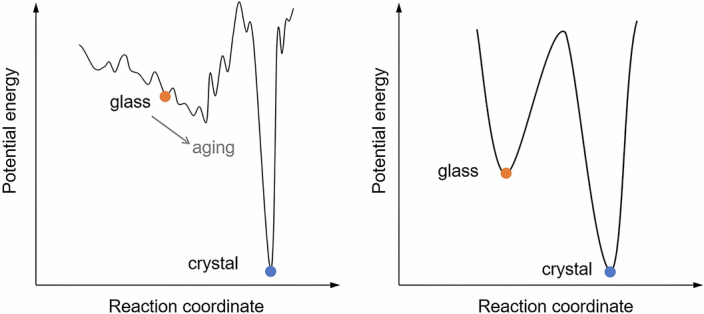
Schematic of the potential energy landscape. A substantial difference is expected for conventional PCMs (left panel, where upon ageing the glass can relax towards a number of possible lower-energy basins) and our new molecule-like PCMs (right panel, in which the glass sits at the bottom of a rather deep basin, while comparable/competing metastable basins are either non-existent, or all out of the picture because they are inaccessible due to high barriers for the glass aged in this study at temperatures below ~200 °C). See text for additional explanation.

We have designed an unconventional PCM without resistance drift at almost all practical IMC operating temperatures (*T* = −200 °C to 165 °C) from the start (immediately after the amorphous state was generated) to many hours of aging at each *T*. Resorting to a glass with molecule-like motifs opens a new avenue towards ultralow resistance drift (Supplementary Note 3). In contrast with conventional a-PCMs (or other glass categories) that tend to readily relax towards other adjacent sub-basins (variable motif configurations) across a fairly shallow megabasin in the potential energy landscape, the molecular-glass-like a-PCM we designed would stay put in a rather steep basin (Extended Data Fig. 5), with little motivation for relaxation and no other desirable motifs that can be accessed (at least up to ~200 °C). The transformation between amorphous and crystalline CrTe_3_ via the cooperation of compact and robust octahedra also resulted in a desirable combination of excellent amorphous stability and satisfactorily fast crystallization speed. Future work is anticipated to gain a deeper understanding of this alloy at temperatures approaching crystallization via in situ measurements^45,46^ and machine-learning-facilitated molecular dynamics^47^. Also, it is necessary to test the multilevel capacity in nanoscale PCM devices^48^, the endurance beyond million cycles, as well as the device variability of CrTe_3_ in a large crossbar array, before the new PCM can be put into practical use. Nevertheless, we have demonstrated that CrTe_3_ thin films can be produced with wafer-scale homogeneity via a standard sputtering approach, adding no complexity to mass production nor to the programming algorithm. Overall, our no-drift discovery achieves the first intrinsically robust a-PCM, offering a front-end materials solution that has the potential to meet the demanding requirements of high-precision multilevel programming at the operation temperatures for both data-centric and edge-computing platforms.

## Methods

### Synthesis and structural characterizations

CrTe_3_ thin films of ~50−150 nm were deposited on four-inch SiO_2_/Si substrates at RT with a pure Cr target and a pure Te target in a high vacuum via AJA ORION 8 (base pressure, less than ~1 × 10^−5^ Pa). An ~10-nm-thick ZnS–SiO_2_ capping layer was grown on top of the CrTe_3_ film inside the vacuum chamber to prevent oxidation and evaporation. The deposition rate was set to 9.5 nm min^−1^. The chemical composition of the sputtered films was determined to be approximately Cr_25.4_Te_74.6_ by the energy-dispersive X-ray experiments. The structures of the as-deposited and post-annealed thin films were investigated by XRD with Cu Kα radiation (Bruker D8 ADVANCE). The TEM specimens were prepared by a dual-beam focused-ion-beam system (Helios NanoLab 600i, FEI) with a Ga ion beam operated at 30 kV. A Pt protective layer was deposited above the CrTe_3_ thin film to mitigate potential damage from Ga ions during the focused-ion-beam lift-out and thinning processes. The TEM experiments were performed on a Talos-F200X device operated at 200 kV. The scanning transmission electron microscopy–high-angle annular dark field imaging and energy-dispersive X-ray mapping experiments were performed on a JEOL ARM200F STEM device with a probe aberration corrector, operated at 200 kV. Raman spectra were collected using Renishaw inVia Qontor with a solid-state 532-nm laser for excitation, where the laser power was set as 0.25 mW, and an exposure time of 2 s with 50 cycles was used.

### Fabrication of devices and the vehicle

The confined memory devices were fabricated using electron-beam lithography and reactive ion etching. The top and bottom electrodes were made of tungsten, and SiO_2_ was used for the dielectric layers. The thickness of the CrTe_3_ layer was ~100 nm. Two lithography processes were adopted for the fabrication of the bridge-like devices. The first lithography process was applied to pattern the layer of a metal electrode on a SiO_2_ wafer using an ultraviolet lithography machine (SUSS MJB4). The tungsten electrodes of 30-nm thickness were deposited on the patterned substrate via magnetron sputtering followed by a lift-out process. The second lithography process was applied to pattern the PCM layer. The deposited CrTe_3_ layer (~50 nm) was slightly thicker than that of the tungsten electrodes to ensure the connectivity of the device. A ZnS–SiO_2_ protective layer with a thickness of 20 nm was deposited on top of the surface. Both lithography processes were done using a negative photoresist (AZ5214E). The 2 × 2 CrTe_3_ device array was fabricated and integrated on a custom printed circuit board using a wire bonder (TPT HB10). The vehicle consisted of two greyscale sensors, a CrTe_3_ array, amplifiers and a motor system (including a servomotor and an engine), batteries and the vehicle body. The servomotor performed different steering actions directed by the CrTe_3_-based control system.

### Electrical measurements

The custom-made electrical testing system was constructed by connecting a Keithley 2636B source meter and an Instec mK200 hot stage with a temperature accuracy of 0.001 °C. The sheet resistance of the CrTe_3_ films as a function of temperature was measured at a heating rate of 10 °C min^−1^ under argon protection. The probe electrodes were made of tungsten. For the electrical measurements of 34 units on the wafer, we first broke down these units from the wafer, and then repeated the sheet resistance measurement on each of them. The physical property measurement system instrument (Quantum Design DynaCool) was used for the Hall effect measurement at RT and the low-temperature resistance measurements between −50 °C to −200 °C. The electrical measurements of the confined memory devices were performed using the Keithley 2400C source meter (measuring cell resistance) and the Tektronix AWG5002B pulse generator (generating voltage pulse). Long voltage pules were used in the cycling experiments to guarantee the devices to form full RESET (2.5 V, 300 ns followed by a 40-ns falling edge) and SET (1.5 V, 500 ns followed by an 80-ns falling edge) states for the subsequent resistance drift measurements. The self-built opto-electro-thermal platform was equipped with Stradus 488–25 lasers, arbitrary function generator (AFG 31000 SERIES), optical path system, Keithley 2636B source meter, Instec mK200 hot stage and a computer. The wavelength, pulse duration and power of the applied laser were set as 488 nm, 100 ns and 90 mW, respectively, for the step-wise crystallization of bridge-like devices. The source meter records the resistance values continuously so that any change in resistance can be monitored in real time on multilevel programming. Slight numerical difference in the electrical resistance value of the amorphous (or crystalline) state is expected for different device setups, in which the film thickness, length, type of electrodes, amorphous-to-crystal ratio and orientation of the crystal can lead to some resistance variations.

### Ab initio calculations

We carried out DFT-based AIMD simulations using the second-generation Car–Parrinello molecular dynamics scheme^49^ implemented using the CP2K package^50^. The Kohn–Sham orbitals were expanded by a double- and triple-zeta plus polarization Gaussian-type basis set for Cr and Te, and the charge density was expanded in plane waves with a cut-off of 300 Ry. The Goedecker–Teter–Hutter pseudopotentials^51^ and Perdew–Burke–Ernzerhof functional^52^ with van der Waals correction based on the Grimme’s D3 method^53^ were applied, and spin polarization was considered using α and β orbitals without spin restriction. The AIMD calculations were carried out using the canonical (*NVT*) ensemble with a time step of 2 fs. To generate a-CrTe_3_, 50 Cr atoms and 150 Te atoms were randomly arranged in a cubic cell. The model was heated above 3,000 K to remove all possible crystalline orders, and were then quenched down to ~1,200 K in 10 ps. After an AIMD run at ~1,200 K for 30 ps, the model was then quenched down to 0 K at a cooling rate of 12.5 K ps^−1^. To collect structural data at different holding temperatures, the corresponding snapshots within the cooling process were picked out for a separate 50-ps AIMD run at the respective holding temperature. Three amorphous models with independent thermal history were calculated in both crystalline and amorphous densities, respectively. The structural relaxation and further spin-polarized DOS analyses at 0 K were conducted using the Vienna ab initio simulation package code^54^ with the Perdew–Burke–Ernzerhof functional, van der Waals D3 correction and the projector-augmented wave pseudopotentials^55^. The energy cut-off was set as 450 eV. Each amorphous model contained 50 Cr atoms and 150 Te atoms, and its Brillouin zone was sampled using the Γ point. For c-CrTe_3_, the unit cell contained 8 Cr atoms and 24 Te atoms, and its Brillouin zone was sampled using a 2 × 2 × 4 *k*-point mesh.

## Online content

Any methods, additional references, Nature Portfolio reporting summaries, source data, extended data, supplementary information, acknowledgements, peer review information; details of author contributions and competing interests; and statements of data and code availability are available at 10.1038/s41563-025-02361-0.

## Supplementary information

Supplementary Information Supplementary Notes 1–3, Figs. 1–17 and references.

Supplementary Video 1 Drift measurement of one CrTe_3_ electronic device after RESET operation.

Supplementary Video 2 The video recorded for CrTe_3_ based path-tracking vehicle.

Supplementary Video 3 The video recorded for GST based path-tracking vehicle.

## Data Availability

All data needed to evaluate the conclusions in this paper are included in the Article or the Supplementary Information.

## Code availability

The Vienna ab initio simulation package and CP2K software packages are commercially available at https://www.vasp.at and https://www.cp2k.org (open source), respectively.

## References

[1] Lanza, M. et al. Memristive technologies for data storage, computation, encryption, and radio-frequency communication. *Science* **376**, eabj9979 (2022). 35653464 10.1126/science.abj9979

[2] Ielmini, D. & Wong, H.-S. P. In-memory computing with resistive switching devices. *Nat. Electron.* **1**, 333–343 (2018).

[3] Zhang, W. et al. Edge learning using a fully integrated neuro-inspired memristor chip. *Science* **381**, 1205–1211 (2023). 37708281 10.1126/science.ade3483

[4] Ambrogio, S. et al. An analog-AI chip for energy-efficient speech recognition and transcription. *Nature* **620**, 768–775 (2023). 37612392 10.1038/s41586-023-06337-5 PMC10447234

[5] Rao, M. et al. Thousands of conductance levels in memristors integrated on CMOS. *Nature* **615**, 823–829 (2023). 36991190 10.1038/s41586-023-05759-5

[6] Wang, Z. et al. Resistive switching materials for information processing. *Nat. Rev. Mater.* **5**, 173–195 (2020).

[7] Wang, M. et al. Robust memristors based on layered two-dimensional materials. *Nat. Electron.* **1**, 130–136 (2018).

[8] Boybat, I. et al. Temperature sensitivity of analog in-memory computing using phase-change memory. In *Proc. IEEE International Electron Devices Meeting* 28.3.1–28.3.4 (IEEE, 2021).

[9] Jiang, H. et al. Sub-10 nm Ta channel responsible for superior performance of a HfO_2_ memristor. *Sci. Rep.* **6**, 28525 (2016). 27334443 10.1038/srep28525 PMC4917839

[10] Wuttig, M. & Yamada, N. Phase-change materials for rewriteable data storage. *Nat. Mater.* **6**, 824–832 (2007). 17972937 10.1038/nmat2009

[11] Zhang, W., Mazzarello, R., Wuttig, M. & Ma, E. Designing crystallization in phase-change materials for universal memory and neuro-inspired computing. *Nat. Rev. Mater.* **4**, 150–168 (2019).

[12] Cheng, H. Y., Carta, F., Chien, W. C., Lung, H. L. & BrightSky, M. 3D cross-point phase-change memory for storage-class memory. *J. Phys. D: Appl. Phys.* **52**, 473002 (2019).

[13] Song, Z. T. et al. High endurance phase change memory chip implemented based on carbon-doped Ge_2_Sb_2_Te_5_ in 40 nm node for embedded application. In *Proc. IEEE International Electron Devices Meeting* 27.5.1–27.5.4 (IEEE, 2018).

[14] Grossier, N. et al. ASIL-D automotive-grade microcontroller in 28 nm FD-SOI with full-OTA capable 21 MB embedded PCM memory and highly scalable power management. In *Proc. IEEE Symposium on VLSI Technology* 1–2 (IEEE, 2023).

[15] Kim, H. J. et al. Versatile spaceborne photonics with chalcogenide phase-change materials. *npj Microgravity* **10**, 20 (2024). 38378811 10.1038/s41526-024-00358-8 PMC10879159

[16] Pirovano, A. et al. Low-field amorphous state resistance and threshold voltage drift in chalcogenide materials. *IEEE Trans. Electron Dev.* **51**, 714–719 (2004).

[17] Boniardi, M. et al. A physics-based model of electrical conduction decrease with time in amorphous Ge_2_Sb_2_Te_5_. *J. Appl. Phys.* **105**, 084506 (2009).

[18] Zhang, W. & Ma, E. Unveiling the structural origin to control resistance drift in phase-change memory materials. *Mater. Today* **41**, 156–176 (2020).

[19] Le Gallo, M. et al. Mixed-precision in-memory computing. *Nat. Electron.* **1**, 246–253 (2018).

[20] Le Gallo, M. et al. Precision of bit slicing with in-memory computing based on analog phase-change memory crossbars. *Neuromorph. Comput. Eng.* **2**, 014009 (2022).

[21] Ghazi Sarwat, S. et al. Projected mushroom type phase-change memory. *Adv. Funct. Mater.* **31**, 2106547 (2021).

[22] Ambrogio, S. et al. Equivalent-accuracy accelerated neural network training using analogue memory. *Nature* **558**, 60–67 (2018). 29875487 10.1038/s41586-018-0180-5

[23] Li, C. et al. Understanding phase-change materials with unexpectedly low resistance drift for phase-change memories. *J. Mater. Chem. C* **6**, 3387–3394 (2018).

[24] Khan, R. S., Dirisaglik, F., Gokirmak, A. & Silva, H. Resistance drift in Ge_2_Sb_2_Te_5_ phase change memory line cells at low temperatures and its response to photoexcitation. *Appl. Phys. Lett.* **116**, 253501 (2020).

[25] Wang, Q. et al. Reliable Ge_2_Sb_2_Te_5_ based phase-change electronic synapses using carbon doping and programmed pulses. *J. Materiomics* **8**, 382–391 (2022).

[26] Ding, K. et al. Phase-change heterostructure enables ultralow noise and drift for memory operation. *Science* **366**, 210–215 (2019). 31439757 10.1126/science.aay0291

[27] Liu, B. et al. Multi-level phase-change memory with ultralow power consumption and resistance drift. *Sci. Bull.* **66**, 2217–2224 (2021). 10.1016/j.scib.2021.07.018 36654113

[28] Hatayama, S., Song, Y.-H. & Sutou, Y. Low resistance-drift characteristics in Cr_2_Ge_2_Te_6_-based phase change memory devices with a high-resistance crystalline phase. *Mater. Sci. Semicond. Process.* **133**, 105961 (2021).

[29] Chen, B. et al. Suppressing structural relaxation in nanoscale antimony to enable ultralow-drift phase-change memory applications. *Adv. Sci.* **10**, 2301043 (2023). 10.1002/advs.202301043 PMC10477879 37377084

[30] Raty, J.-Y. et al. Aging mechanisms of amorphous phase-change materials. *Nat. Commun.* **6**, 7467 (2015). 26105012 10.1038/ncomms8467

[31] Gabardi, S., Caravati, S., Sosso, G. C., Behler, J. & Bernasconi, M. Microscopic origin of resistance drift in the amorphous state of the phase-change compound GeTe. *Phys. Rev. B* **92**, 054201 (2015).

[32] Konstantinou, K., Mocanu, F. C., Lee, T. H. & Elliott, S. R. Revealing the intrinsic nature of the mid-gap defects in amorphous Ge_2_Sb_2_Te_5_. *Nat. Commun.* **10**, 3065 (2019). 31296874 10.1038/s41467-019-10980-w PMC6624207

[33] McGuire, M. A. et al. Antiferromagnetism in the van der Waals layered spin-lozenge semiconductor CrTe_3_. *Phys. Rev. B* **95**, 144421 (2017).

[34] Hatayama, S. et al. Inverse resistance change Cr_2_Ge_2_Te_6_-based PCRAM enabling ultralow-energy amorphization. *ACS Appl. Mater. Interfaces* **10**, 2725–2734 (2018). 29280374 10.1021/acsami.7b16755

[35] Wang, X., et al. Spin glass behavior in amorphous Cr_2_Ge_2_Te_6_ phase-change alloy. *Adv. Sci.* **10**, 2302444 (2023). 10.1002/advs.202302444 PMC10427411 37279377

[36] Njoroge, W. K., Woeltgens, H.-W. & Wuttig, M. Density changes upon crystallization of Ge_2_Sb_2.04_Te_4.74_ films. *J. Vac. Sci. Technol. A* **20**, 230–233 (2002).

[37] Salinga, M. et al. Monatomic phase change memory. *Nat. Mater.* **17**, 681–685 (2018). 29915424 10.1038/s41563-018-0110-9

[38] Wimmer, M., Kaes, M., Dellen, C. & Salinga, M. Role of activation energy in resistance drift of amorphous phase change materials. *Front. Phys.* **2**, 75 (2014).

[39] Navarro, G. et al. Trade-off between SET and data retention performance thanks to innovative materials for phase-change memory. In *Proc. IEEE International Electron Devices Meeting* 21.5.1–21.5.4 (IEEE, 2013).

[40] Zhou, L. et al. Resistance drift suppression utilizing GeTe/Sb_2_Te_3_ superlattice-like phase-change materials. *Adv. Electron. Mater.* **5**, 1900781 (2019).

[41] Khan, A. I. et al. Ultralow–switching current density multilevel phase-change memory on a flexible substrate. *Science* **373**, 1243–1247 (2021). 34516795 10.1126/science.abj1261

[42] Wu, X. et al. Novel nanocomposite-superlattices for low energy and high stability nanoscale phase-change memory. *Nat. Commun.* **15**, 13 (2024). 38253559 10.1038/s41467-023-42792-4 PMC10803317

[43] Burr, G. W. et al. Large-scale neural networks implemented with non-volatile memory as the synaptic weight element: comparative performance analysis (accuracy, speed, and power). In *Proc. IEEE International Electron Devices Meeting* 4.4.1–4.4.4 (IEEE, 2015).

[44] Wang, C. et al. A Braitenberg vehicle based on memristive neuromorphic circuits. *Adv. Intell. Syst.* **2**, 1900103 (2019).

[45] Zalden, P. et al. Femtosecond X-ray diffraction reveals a liquid-liquid phase transition in phase-change materials. *Science* **364**, 1062–1067 (2019). 31197008 10.1126/science.aaw1773

[46] Jiang, T.-T. et al. In situ characterization of vacancy ordering in Ge-Sb-Te phase-change memory alloys. *Fundam. Res.* **4**, 1235–1242 (2024). 39431143 10.1016/j.fmre.2022.09.010 PMC11489497

[47] Zhou, Y., Zhang, W., Ma, E. & Deringer, V. L. Device-scale atomistic modelling of phase-change memory materials. *Nat. Electron.* **6**, 746–754 (2023).

[48] Kim, W. et al. Confined PCM-based analog synaptic devices offering low resistance-drift and 1000 programmable states for deep learning. In *Proc. IEEE Symposium on VLSI Technology* T66–T67 (IEEE, 2019).

[49] Kühne, T., Krack, M., Mohamed, F. & Parrinello, M. Efficient and accurate car-parrinello-like approach to Born-Oppenheimer molecular dynamics. *Phys. Rev. Lett.* **98**, 066401 (2007). 17358962 10.1103/PhysRevLett.98.066401

[50] Kühne, T. D. et al. CP2K: an electronic structure and molecular dynamics software package—quickstep: efficient and accurate electronic structure calculations. *J. Chem. Phys.* **152**, 194103 (2020). 33687235 10.1063/5.0007045

[51] Goedecker, S., Teter, M. & Hutter, J. Separable dual-space Gaussian pseudopotentials. *Phys. Rev. B* **54**, 1703 (1996). 10.1103/physrevb.54.1703 9986014

[52] Perdew, J. P., Burke, K. & Ernzerhof, M. Generalized gradient approximation made simple. *Phys. Rev. Lett.* **77**, 3865–3868 (1996). 10062328 10.1103/PhysRevLett.77.3865

[53] Grimme, S., Antony, J., Ehrlich, S. & Krieg, H. A consistent and accurate ab initio parametrization of density functional dispersion correction (DFT-D) for the 94 elements H-P. *J. Chem. Phys.* **132**, 154104 (2010). 20423165 10.1063/1.3382344

[54] Kresse, G. & Furthmüller, J. Efficient iterative schemes for ab initio total-energy calculations using a plane-wave basis set. *Phys. Rev. B* **54**, 11169 (1996). 10.1103/physrevb.54.11169 9984901

[55] Blöchl, P. E. Projector augmented-wave method. *Phys. Rev. B* **50**, 17953–17979 (1994). 10.1103/physrevb.50.17953 9976227

