# Supplementary material for: Amorphous phase-change memory alloy with no resistance drift
Source: Nat Mater. 2025 Oct 1;25(3):456–62. doi: 10.1038/s41563-025-02361-0 (PMC12960214; doi:10.1038/s41563-025-02361-0)
Supplement: Supplementary file 1 — Supplementary Notes 1–3, Figs. 1–17 and references. [file 41563_2025_2361_MOESM1_ESM.pdf]

nature materials

Supplementary information https://doi.org/10.1038/s41563-025-02361-0

Amorphous phase-change memory alloy with no resistance drift

In the format provided by the authors and unedited

Table of contents

Supplementary Notes 1-3

Supplementary Reference

Supplementary Figures 1-17

Supplementary Note 1

We first noticed that Peierls distortion is nearly non-existent in all crystalline polymorphs of 3*d* transition metal (TM) tellurides at Te-rich compositions (Te:TM ≥ 2:1), where each 3*d* transition metal atom is coordinated by six Te atoms, forming an octahedral motif with symmetric bonding along each direction. Although the octahedral motifs can be slightly distorted, with the Te-TM-Te bond angle being slightly smaller or larger than 90°, there is no long/short bond correlation to establish *bona fide* Peierls distortion (see Supplementary Fig. S1). We conjectured that this scenario could be retained to the amorphous counterpart of such tellurides. Given the Te-rich composition, the probability to form wrong (TM-TM) bonds in the amorphous state is also reduced. It is then promising that the two factors (i) and (ii) in the preceding paragraph can both be minimized. To this end, we scrutinized all 3*d* transition metal tellurides. Among them, $CrTe_3$ is the richest in Te composition.

Supplementary Note 2

To assess the crystallization capacity of $CrTe_3$, we used laser pulse of 100 ns (90 mW) to generate crystallized a-$CrTe_3$ domains, as confirmed by Raman spectroscopy measurements in Supplementary Fig. S14a. This crystallization speed may seem surprisingly fast, considering the a-$CrTe_3$ is rather stable against crystallization with a high activation energy for crystallization, E_a ~3.28 eV (Supplementary Fig. S14b, higher than the 2.2 eV known for GST). This is because fast crystal growth in $CrTe_3$ could proceed via relatively quick rearrangement of [$CrTe_6$] octahedra, given sufficient thermal energy, as the Cr and Te atoms still move collectively, i.e., the robust octahedra do not fall apart into individual atoms that diffuse independently (see AIMD calculations at 300 and 400 °C in Supplementary Fig. S14c).

Supplementary Note 3

The new a-PCM is characterized by "molecule-like" motifs that are unique in configuration, overwhelmingly predominant throughout the structure, and robust over time. To explore the general validity of such a structural feature, we carried out AIMD simulations and electrical measurement of amorphous $TiTe_2$ and VTe_2. As shown in Supplementary Fig. S16, the molecule-like motifs of [$TiTe_6$] and [VTe_6] octahedra also prevailed, and the measured drift coefficient of a-$TiTe_2$ and a-VTe_2 thin

films was as small as that of a-$CrTe_3$. Nevertheless, the limited contrast window and the low crystallization temperature of the two alloys render them unfit for practical applications. We also note that not all Te-rich transition metal tellurides should be regarded as molecule-like PCMs. In a very recent work [S1], a low-drift behavior was found for a-$NbTe_4$ thin film at room temperature. But no clear molecule-like pattern was observed in the a-$NbTe_4$ amorphous model obtained via AIMD simulations. Indeed, this low-drift behavior cannot be sustained at slightly elevated temperatures: the drift coefficient quickly increased to ~0.082 at 80 °C (see Supplementary Fig. S17).

Supplementary References

S1. Shuang, Y. *et al.* $NbTe_4$ Phase-Change Material: Breaking the Phase-Change Temperature Balance in 2D Van der Waals Transition-Metal Binary Chalcogenide. *Adv. Mater.* **35**, 2303646 (2023).

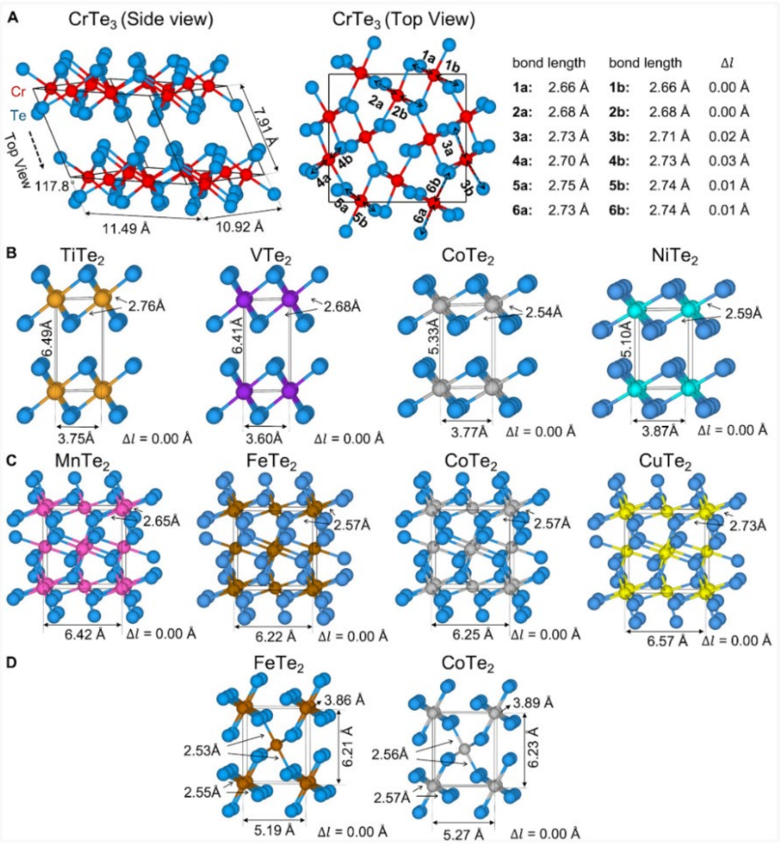

Supplementary Figure 1. The DFT-optimized crystal structures of the Te-rich 3d-transition-metal tellurides found in the inorganic crystal structure database (ICSD). (a) Monoclinic $CrTe_3$. The bonds along each direction were marked. (b) Hexagonal $TiTe_2$, VTe_2, $CoTe_2$ and $NiTe_2$. (c) Cubic $MnTe_2$, $FeTe_2$, $CoTe_2$ and $CuTe_2$. (d) Tetragonal $FeTe_2$ and $CoTe_2$.

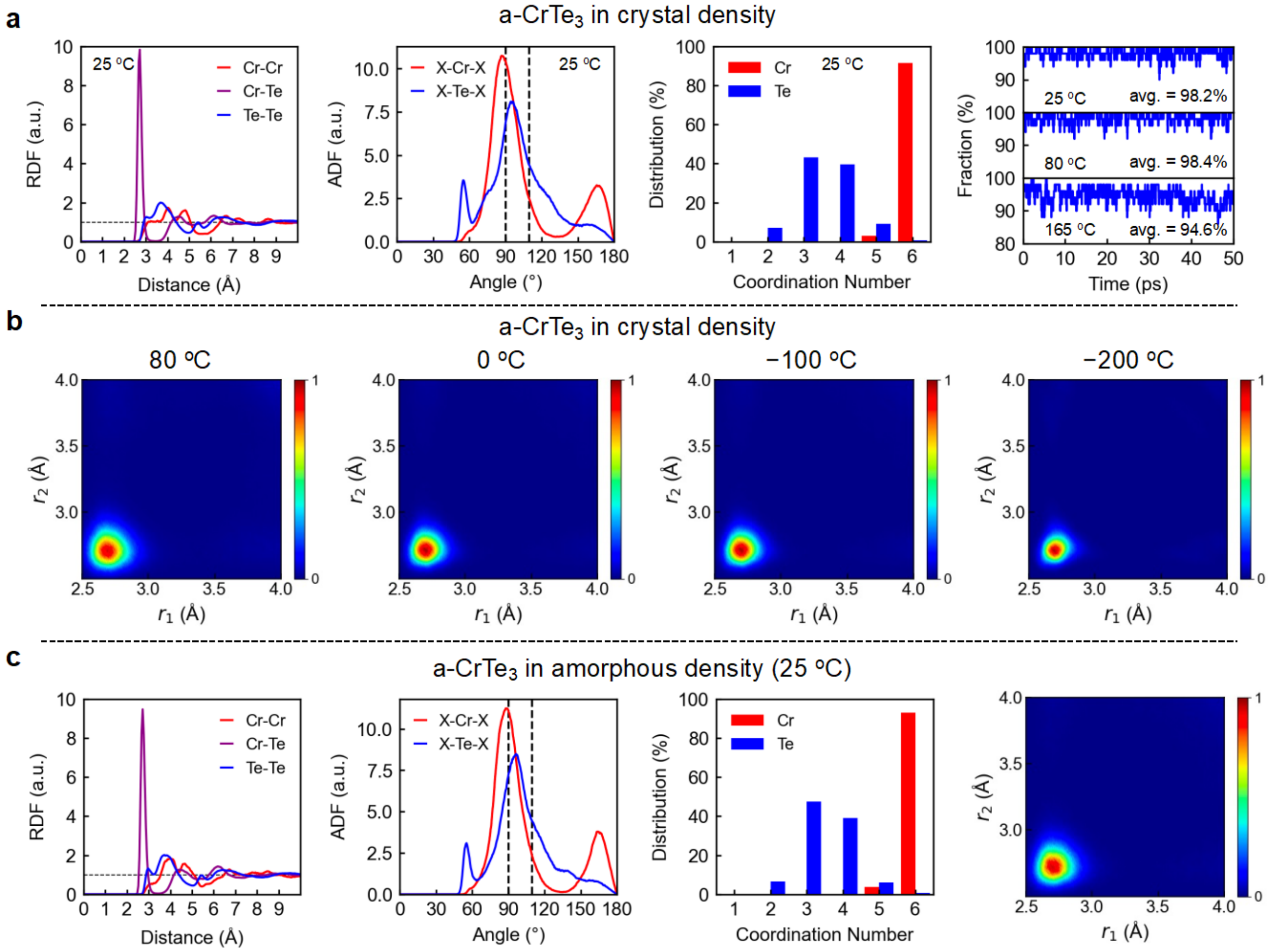

Supplementary Figure 2. Structural analyses of a-$CrTe_3$ models at different temperatures. The interatomic cutoffs of 2.9, 3.4 and 3.2 Å were used for Cr-Cr, Cr-Te, and Te-Te, respectively. (a) The radial distribution function (RDF), angular distribution function (ADF), the distribution of coordination number (CN), and changes in number density of $[CrTe_6]$ octahedra over time of the a-$CrTe_3$ model in crystal density. The number of $[CrTe_6]$ octahedron was determined by calculating the bond order parameter $q = 1 - 3/8\sum_{x>y}(1/3 + cos\theta_{xmy})$, where θ_{xmy} represents the bond angle of the center atom m with its two neighboring atoms x and y. The fraction of $[CrTe_6]$ octahedra at 165 °C can be increased, if the Cr-Te cutoff was increased to account for the stronger thermal vibrations at such T. (b) The ALTBC plots of the a-$CrTe_3$ model in crystal density calculated at 80°C, 0°C, –100°C and –200°C. (c) The RDF, ADF, CN distribution, and ALTBC of $CrTe_3$ models in amorphous density calculated at 25°C.

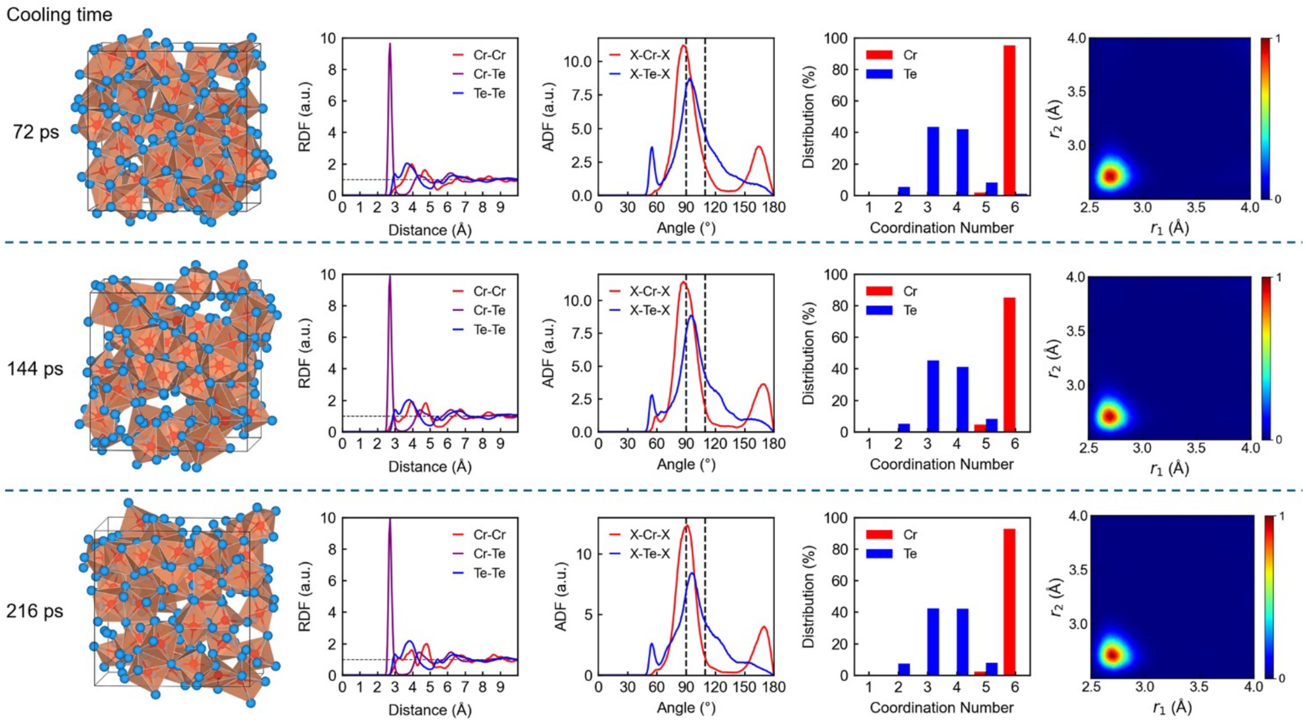

Supplementary Figure 3. The molecule-like structural features of a-$CrTe_3$ remained basically the same upon cooling from 1200 K to 300 K with longer cooling time, namely, 144 ps and 216 ps as compared to 72 ps (used in Figure 1).

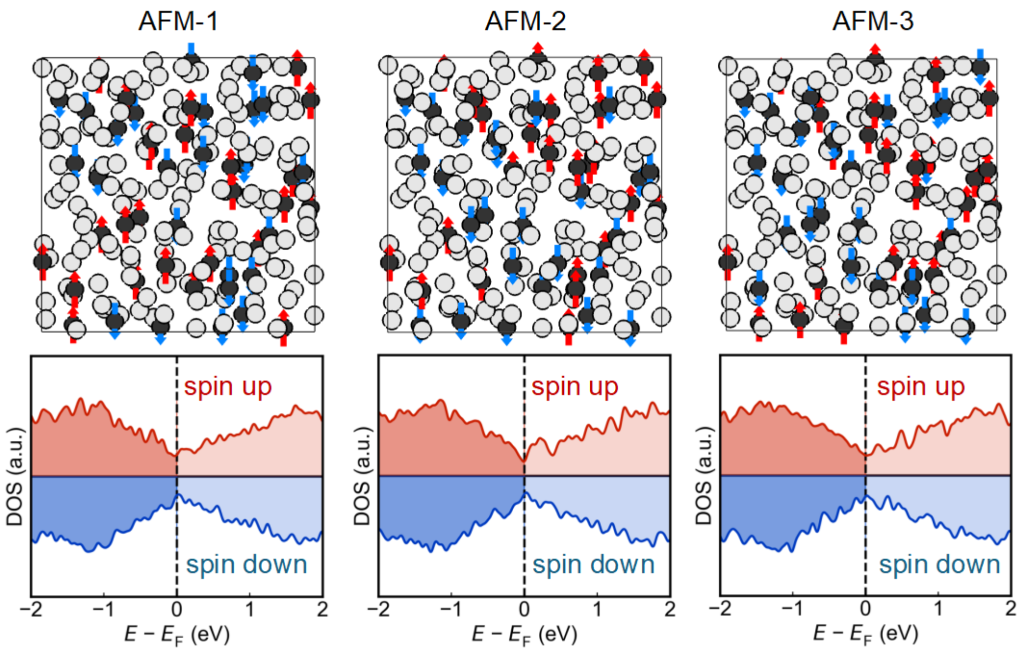

Supplementary Figure 4. The a-$CrTe_3$ model calculated in three antiferromagnetic configurations (equal amount of Cr atoms assigned with spin up and spin down moments). The three a-$CrTe_3$ models were further relaxed. The Cr and Te atoms are rendered in black and gray circles. The red and blue arrows mark the Cr atomic moment directions, respectively. These models were ~5.2 meV/atom higher in energy than the ferromagnetic a-$CrTe_3$ model, and ~106.2 meV/atom higher than the antiferromagnetic c-$CrTe_3$ model, shown in Fig. 1.

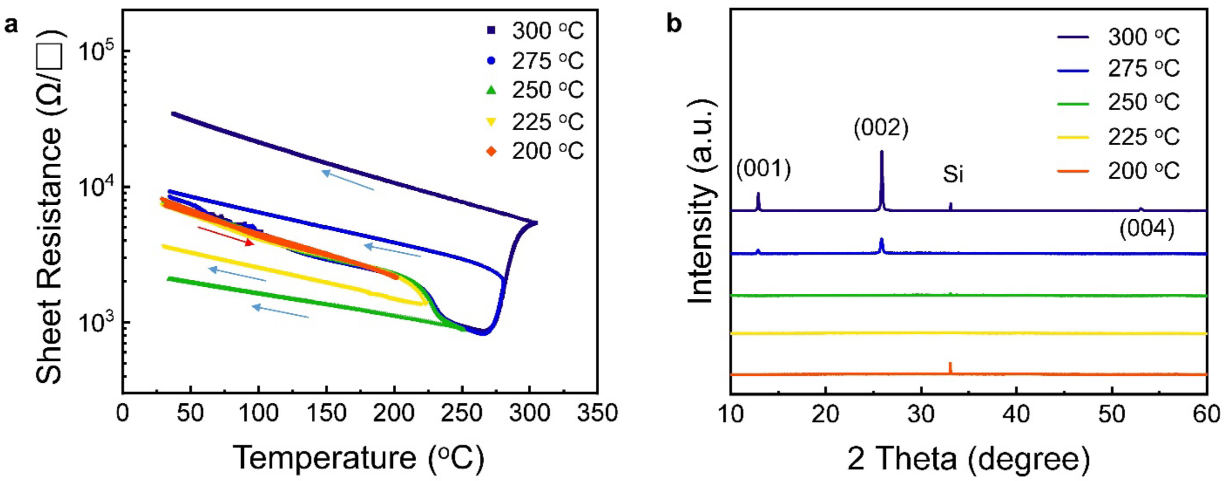

Supplementary Figure 5. (a) The *R*-T electrical measurements and (b) the XRD characterizations on $CrTe_3$ thin film samples heated to different temperatures. Starting from the as-deposited amorphous phase, we heated the samples to 200, 225, 250, 275 and 300 °C, respectively. The sample heated to 200 °C showed no visible annealing effect and the initial amorphous phase remained intact. The two samples heated to 275 and 300°C were crystallized, but their resistance values at RT are smaller than that of the 350 °C annealed sample due to their lower degree of crystallinity, as evidenced by the intensity of the XRD peaks in (b). For the samples heated to 225 and 250 °C, clear annealing effects were observed in the amorphous structure, resulting in lower resistance values upon cooling to RT. The resistance drift data reported in this paper were exclusively for temperatures below 200 °C, which already cover a sufficiently wide range for practical applications. The change in the amorphous structure at high temperatures (225 and 250 °C) prior to crystallization requires a separate study but is beyond the scope of this paper.

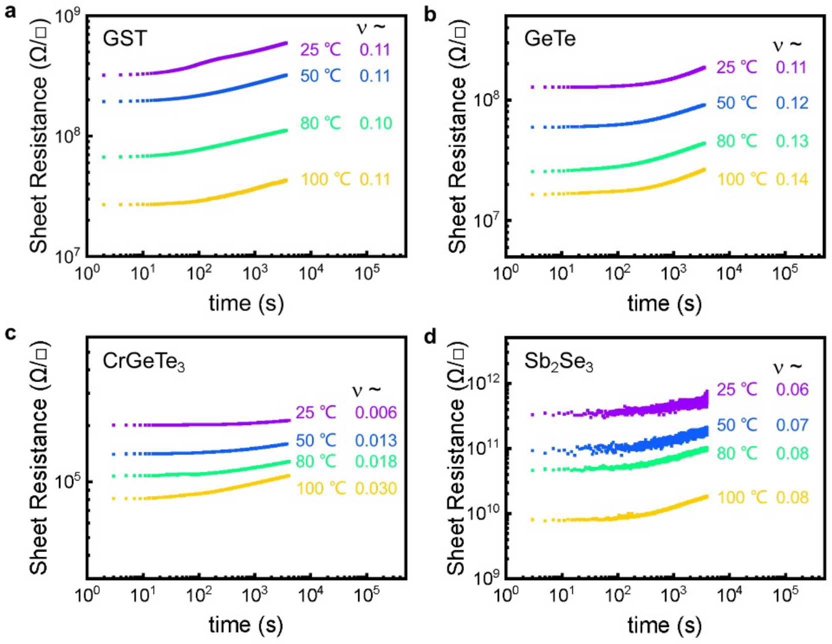

Supplementary Figure 6. To compare with our a-$CrTe_3$, resistance drift tests were performed on (a) a-GST, (b) a-GeTe, (c) a-$CrGeTe_3$ and (d) a-Sb_2Se_3 thin films, each respectively at 25, 50, 80 and 100°C. Significant fluctuations were observed in a-Sb_2Se_3 thin films (at 25 and 50 °C), because their high resistance values approached the measurement limit of our setup.

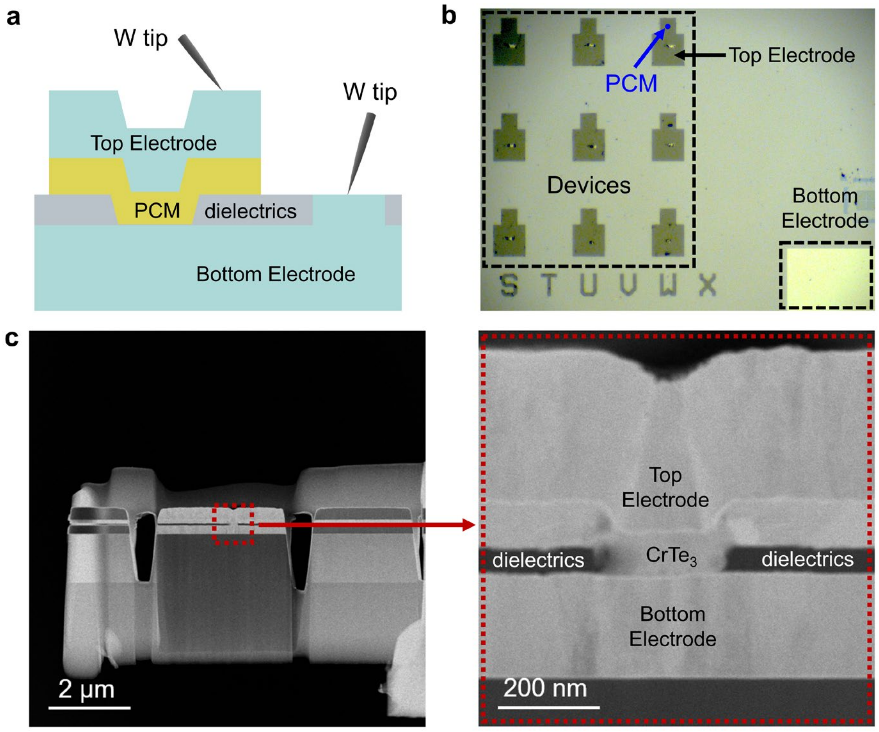

Supplementary Figure 7. (a) The schematic diagram of electrical measurements. (b) The optical image of several $CrTe_3$ electronic devices. (c) The cross-sectional STEM images of one $CrTe_3$ confined memory device. The overall cell structure and the local cell unit are shown in the left and right figure panel. The top and bottom electrodes are made of tungsten and the dielectric layers are SiO_2.

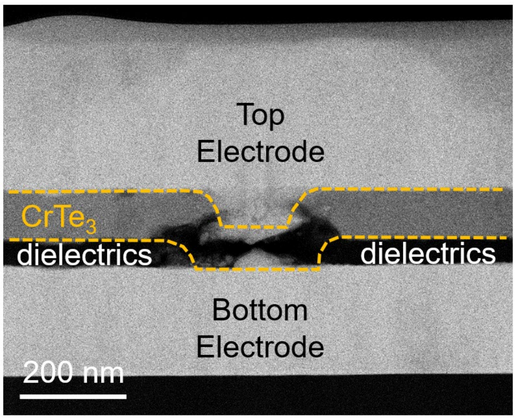

Supplementary Figure 8. One $CrTe_3$ confined memory cell was cycled over 2.4×10^5 times, and the device got stuck in the high resistance state. The cross-sectional STEM analysis of the failed device showed that the tungsten electrodes and the SiO_2 dielectric confinement layers were severely deformed and occupied the programming area of $CrTe_3$, which could be attributed to the limited processing quality of the devices.

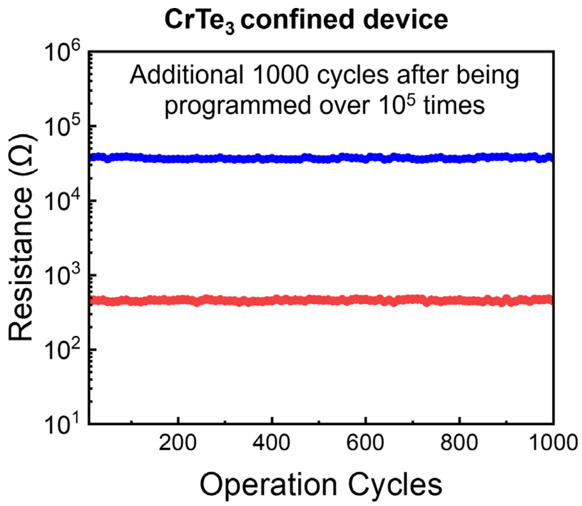

Supplementary Figure 9. Starting from one $CrTe_3$ device that had been programmed 10^5 times, we performed additional switching experiment for another 1,000 cycles. By increasing the pulse width to 800 ns, the programming noise was suppressed.

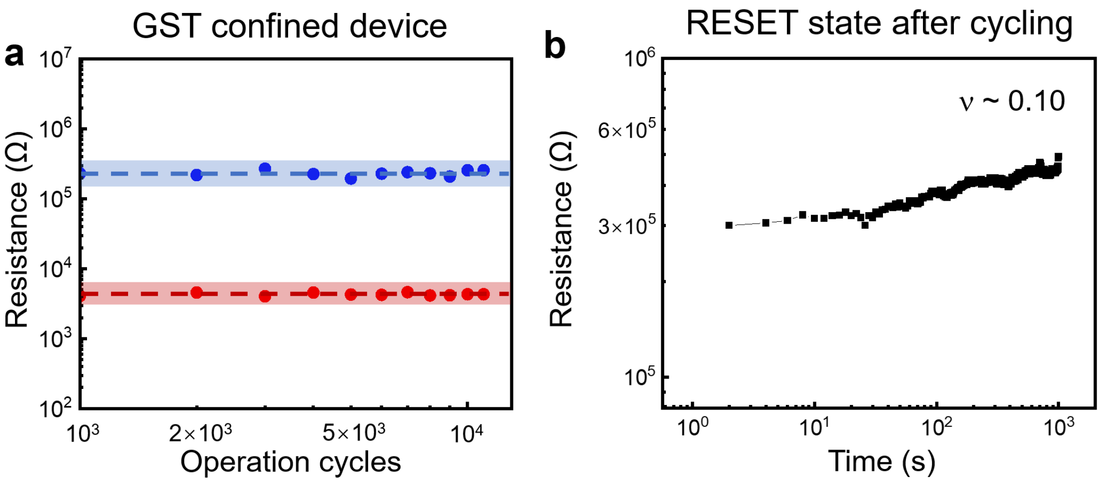

Supplementary Figure 10. (a) The cycling experiment of one GST device. (b) After programming this device over 10^4 times, the device was kept at the high-resistance amorphous state for drift measurement. Even after this extended cycling, the relaxation was still rampant and the measured drift coefficient was still as high as ~0.1 at RT, similar to that of the initial as-deposited amorphous GST thin film.

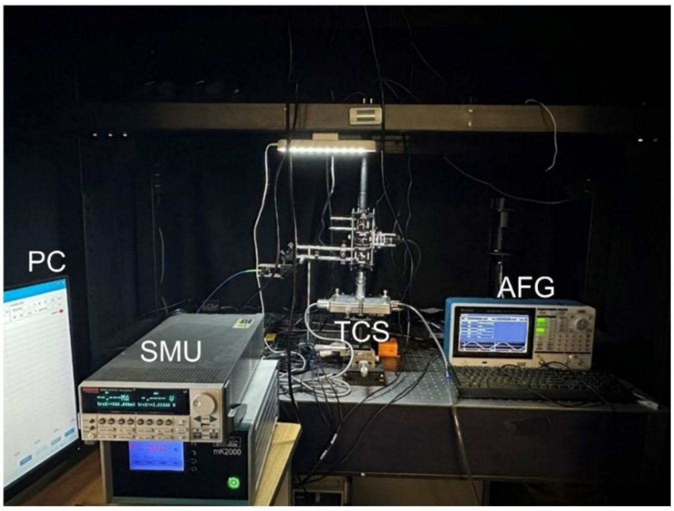

Supplementary Figure 11. The photo of the self-built opto-electro-thermal testing platform.

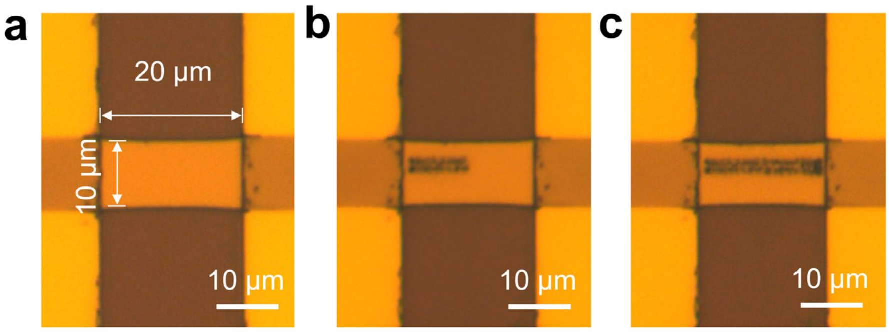

Supplementary Figure 12. The optical images of the bridge-like devices with l = 20 μm and d = 10 μm. (a) The initial device was covered with as-deposited amorphous $CrTe_3$ thin film. (b-c) The device in partially crystallized states. The dark domains indicate that parts of the film are switched to the crystalline phase.

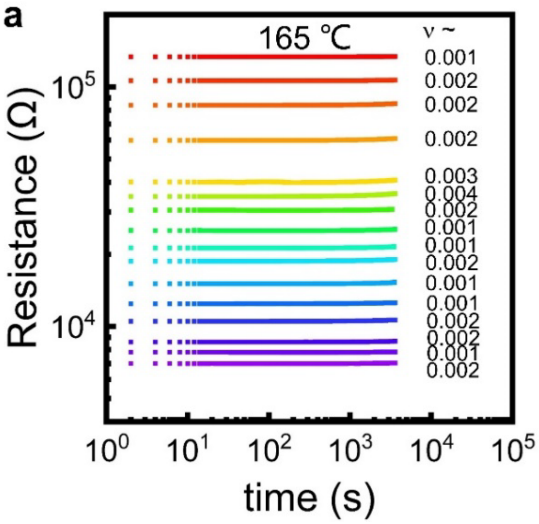

Supplementary Figure 13. The 16 resistance states obtained using the small bridge-like device, showing that at 165 °C the maximum drift coefficient was ~0.004.

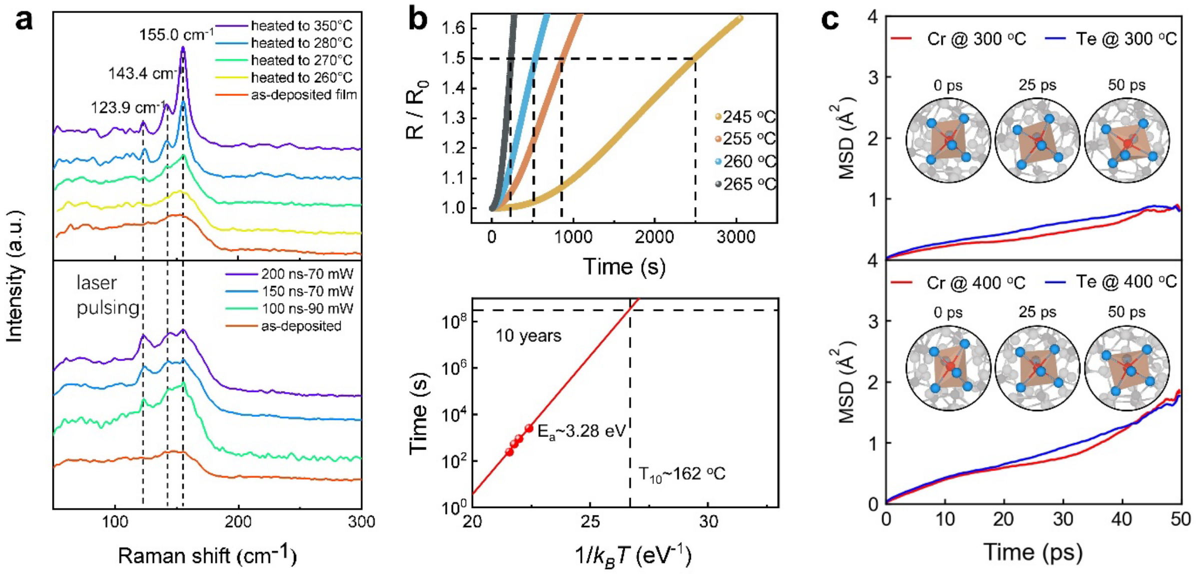

Supplementary Figure 14. Switching and dynamics. (a) The measured Raman spectrum of the as-deposited film, and the films being heated to 260 °C, 270 °C, 280 °C and 350 °C, respectively (upper panel). The Raman spectrum measured at the laser-irradiated spots of the films (lower panel). (b) The electrical resistance of as-deposited $CrTe_3$ films measured at 245 °C, 255 °C, 260 °C and 265 °C, respectively (upper panel). The extrapolated 10-year data retention temperature $T_{10\text{-year}}$ and the estimated activation energy E_a of crystallization for a-$CrTe_3$ (lower panel). (c) The mean squared displacement (MSD) curves of a-$CrTe_3$ at 300°C and 400°C via AIMD calculations. The insets show a series of snapshots of a typical [$CrTe_6$] octahedron taken at 0, 25 and 50 ps of the AIMD trajectories at respective temperatures. The [$CrTe_6$] octahedron was robust, and the associated 1 Cr atom and 6 Te atoms moved collectively. At both holding temperatures, the fraction of Cr atoms in [$CrTe_6$] octahedron is over 90% despite of thermal fluctuations.

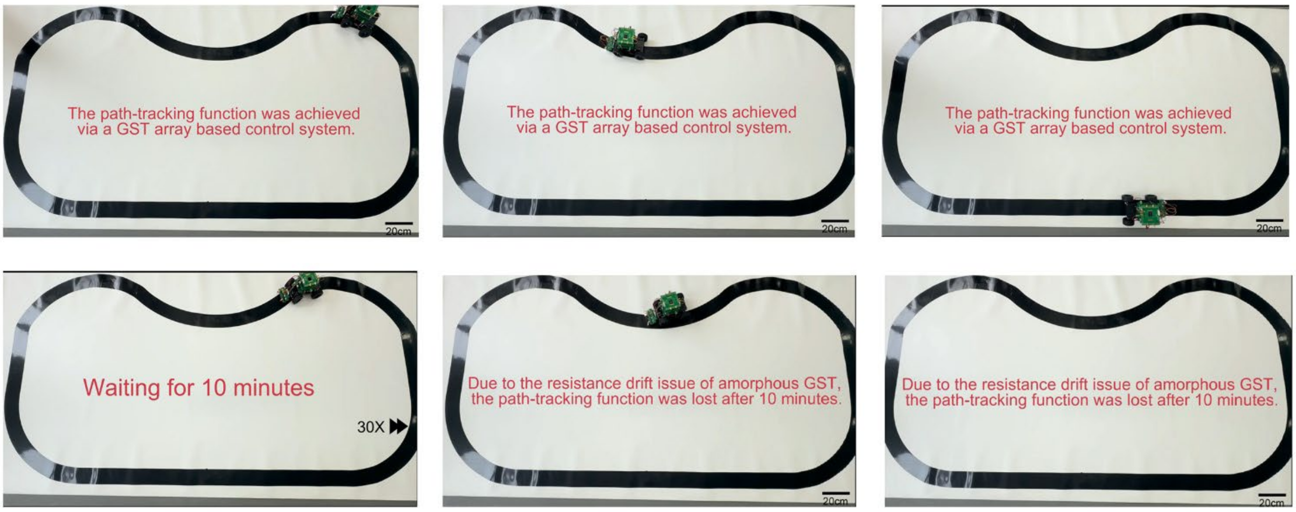

Supplementary Figure 15. The performance of a GST-array based path-tracking vehicle, to contrast with our $CrTe_3$ case. Four GST bridge-like devices were programmed to four separate resistance levels, and the automatic path-tracking function was achieved at this outset. However, only after 10 minutes, the path-tracking function was lost, and the vehicle ran away from the track. See Supplementary Video 3 for the entire test process.

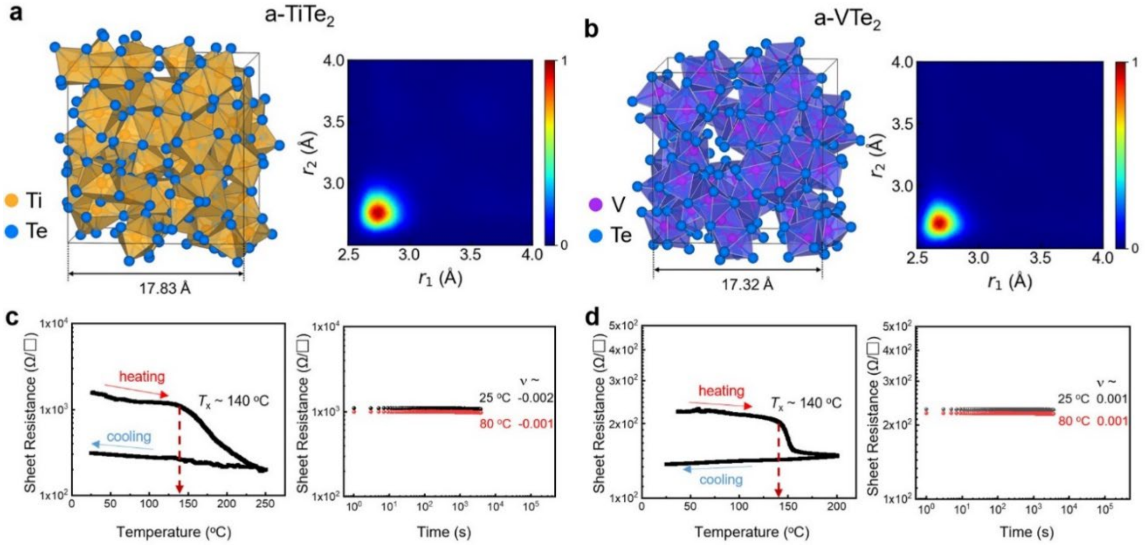

Supplementary Figure 16. The AIMD modeling and electrical measurements of a-$TiTe_2$ and a-VTe_2. (a) A snapshot of a-$TiTe_2$ and the ALTBC plot of a-$TiTe_2$ annealed at 25 ºC. (b) A snapshot of a-VTe_2 and the ALTBC plot of a-VTe_2 annealed at 25 ºC. The Ti, V and Te atoms are rendered with orange, purple and blue spheres. The as-deposited (c) $TiTe_2$ and (d) VTe_2 thin film of ~50 nm both showed a T_x of ~140 °C upon heating. Their drift coefficient values at 25 °C and 80 °C were comparable to those of a-$CrTe_3$ thin films.

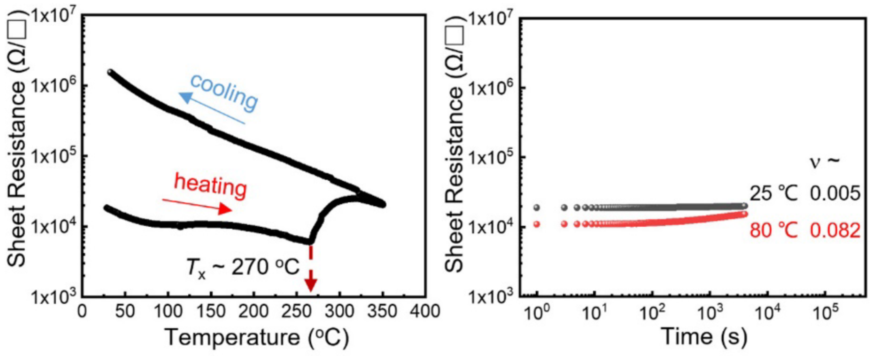

Supplementary Figure 17. Electrical measurements of as-deposited $NbTe_4$ thin films of ~50 nm. The R-T curve showed an inverse contrast window upon crystallization. The drift coefficient ν of a-$NbTe_4$ thin film was measured to be ~0.005 at 25 °C, markedly increasing to ~0.082 at 80 °C.
